# Daidzein Prevents the Increase in CD4^+^CD28null T Cells and B Lymphopoesis in Ovariectomized Mice: A Key Mechanism for Anti-Osteoclastogenic Effect

**DOI:** 10.1371/journal.pone.0021216

**Published:** 2011-06-22

**Authors:** Abdul Malik Tyagi, Kamini Srivastava, Kunal Sharan, Dinesh Yadav, Rakesh Maurya, Divya Singh

**Affiliations:** 1 Division of Endocrinology, Central Drug Research Institute (Council of Scientific and Industrial Research), Chattar Manzil, Lucknow, India; 2 Division of Medicinal and Process Chemistry, Central Drug Research Institute (Council of Scientific and Industrial Research), Chattar Manzil, Lucknow, India; Kyushu Institute of Technology, Japan

## Abstract

Estrogen deficiency leads to an upregulation of TNF-α producing T cells and B-lymphopoesis which augments osteoclastogenesis. Estrogen deficiency also increases the population of premature senescent CD4^+^CD28null T cells which secrete a higher amount of TNF-α thus leading to enhanced osteoclastogenesis. Isoflavonoids like daidzein and genistein are found mostly in soybeans, legumes, and peas. These share structural similarity with 17β-stradiol (E2) and have osteoprotective role. This study explores the effect of daidzein (Daid) on the proliferation of TNF-α producing T cells, premature senescent T cells and B cell lymphopoesis under estrogen deficient conditions. For this study adult Balb/c mice were treated with Daid at 10 mg/kg body weight dose by oral gavage daily post ovariectomy (Ovx). After six weeks animals were autopsied and bone marrow and spleen cells were collected for FACS analysis. Blood serum was collected for ELISA. It was observed that Ovx mice treated with Daid for six weeks show reduction in Ovx induced expansion of CD4^+^ T cells in bone marrow and spleen when analysed by flow cytometry. Estrogen deficiency led to increased prevalence of TNF-α secreting CD4^+^CD28null T cells, however, treatment with Daid increased the percentage of CD4^+^CD28^+^ T cells. Co-culture of CD4^+^CD28null T cells and bone marrow resulted in enhanced osteoclastogenesis as evident by increased tartarate resistant acid phosphatase (TRAP) expression, an osteoclast marker. However, treatment with Daid resulted in reduced osteoclastogenesis in CD4^+^CD28null T cells and bone marrow cell co-culture. Daid also regulated B lymphopoesis and decreased mRNA levels of RANKL in B220^+^ cells. Taken together, we propose that one of the mechanisms by which Daid prevents bone loss is by reversing the detrimental immune changes as a result of estrogen deficiency.

## Introduction

It is now well recognized that one of the mechanism by which estrogen deficiency leads to increased bone loss is by stimulating osteoclast formation. Studies by Roggia et al. [1] have shown that in the bone wasting induced by estrogen deficiency, activated T cells play an essential causal role. Ovariectomy (Ovx) up regulates the production of TNF (Tumor necrosis factor)-α secreting T cells. The amount of TNF-α produced by T cells of Ovx mice is sufficient to augment receptor activator of nuclear factor kappa-B ligand (RANKL)-induced osteoclastogenesis [1]. Immunophenotypical analyses of peripheral blood lymphocyte reveal that several subsets of T lymphocytes (CD3^+^, CD4^+^ and CD8^+^) are increased in osteoporotic patients [2], [3], [4].

Additionally, there is a steady decline in bio available estrogen in aging men and women, superimposed with decrease in estrogen level in women at menopause. Thus, E2 deficiency signals the onset of senescence. The most evident phenotypic change in cellular senescence is the loss of CD28 [5], [6], a T cell-restricted membrane glycoprotein that provides requisite co-stimulatory signal for the generation of T cell-mediated immune responses [5], [7]. CD28null T cells are thought to be biological indicators of immunosenescence [5]. Although CD28 is constitutively expressed on all T cells, CD28null T cells are typically found in the aging immune system, in both CD4^+^ and CD8^+^ subsets [5], [7], [8], [9], [10]. Also, there is increasing evidence that CD28 null T cells are a common feature of inflammatory conditions like rheumatoid arthritis [5]. Studies in our lab have shown that there is increased prevalence of CD28null T cells in Ovx mice which secrete high amount of TNF-α [11]. Apart from T cells, Ovx selectively stimulates B-lymphopoiesis which results in marked accumulation of B220^+^ B cells in mouse bone marrow [12]. *In vitro* studies have demonstrated that mature B cells have the potential to both positively and negatively impact osteoclastogenesis by virtue of their capacity to secrete pro-osteoclastogenic cytokines including RANKL, as well as anti-osteoclastogenic cytokines such as osteoprotegerin (OPG) and TGF-β [13].

Although E2 supplementation protects against Ovx-induced bone loss, E2 replacement therapy (ERT) is less popular in postmenopausal women because of increased risk of breast and uterine cancers in women taking ERT [14]. A growing body of literature suggest that phytoestrogens may confer substantial benefits to bone health without posing the risk of cancer associated with E2 [15], [16]. Isoflavones including genistein and daidzein make up the most common form of phytoestrogens. These share structural similarity to the estrogen 17ß- estradiol and have been shown to have bone protective effect *in vitro* and *in vivo* [17]. High dietary intake of these isoflavones have been reported to increase BMD in lumbar spine of Japanese [18], Chinese [19] and American [20] postmenopausal women. At micromolar concentrations *in vitro*, genistein and daidzein promote osteoblast functions via estrogen receptor (ER)-dependent mechanism [21]. Oral or subcutaneous injections of genistein and daidzein inhibit bone loss in ovariectomized or orchidectomized mice [22].

Amongst the two isoflavones, genistein has been most widely studied for its effect on immunity. It inhibits the lymphocyte proliferation induced by mitogen and alloantigen *in vitro* [23]. Genistein induces dose-responsive reductions in thymus weight in Ovx mice [24]. Like estrogen, genistein prevented the elevation of B-lymphopoiesis in the bone marrow of Ovx mice [25]. Immune effects of genistein have shown to be both ER dependent and ER independent [23]. However, data on Daid mediated immune effects is very limited. In this study we attempt to provide a possible role of dietary isoflavonoid, Daid in immunity. In an earlier study we found that E2 as well as Medicarpin reduce the Ovx induced proliferation of TNF-α secreting T cells and decrease the prevalence of premature senescent cells producing high amounts of TNF-α post Ovx [11]. Here, we evaluate the immunomodulating effect of Daid at its osteoprotective dose on TNF-α producing T cell proliferation and further, if it inhibits the Ovx induced increase in TNF-α secreting CD4^+^CD28null T cells. We have also determined if these premature senescent cells secreting higher amounts of TNF-α contribute to enhanced osteoclastogenesis and if Daid inhibits this phenomenon. Additionally, we also study the effect of Daid on B-lymphopoesis post Ovx and the cytokines (OPG and RANKL) secreted by B cells.

## Results

### Effect of Daid on Ovx induced gain in thymus and spleen weight at its osteoprotective dose

Mice Ovx for 6 weeks exhibited deterioration of trabecular microarchitecture compared with sham, and Ovx mice treated with Daid (10.0 mg kg^−1^.day^−1^) or E2 (0.01 mg.kg^−1^.day^−1^) for 6 weeks protected against Ovx-induced loss of trabecular microarchitecture (Table 1). It is well known that Ovx leads to an increase in thymic weight and cellularity, and E2 administration causes thymic atrophy [26]. As expected, there was increase in thymus weight in Ovx mice (Table 2). In addition, Ovx also induces gain in spleen weight [27]. However, treatment with Daid at its osteoprotective dose inhibited the Ovx induced increase in thymus and spleen weight (Table 2).

**Table 1:** μ-CT analysis of femur trabeculae of Balb/C Mice.

Paramete	Sham	OVX	OVX+E2 0.01 mg/kg/day	OVX+Daid 10 mg/kg/day
Percent bone volume(BV/TV)	22.7±7.66***	7.34±0.302	18.84±6.88***	9.322±2.36*
Trabecular separationTb.Sp(mm)	0.24±0.03***	0.52±0.12	0.38±0.11***	0.38±0.09**
Trabecular numberTb.N(1/mm)	2.83±0.75***	1.11±0.07	2.19±0.67***	1.39±0.29*
Trabecular pattern factorTb.Pf	10.24±5.72***	18.70±2.9	11.20±4.40***	15.80±2.68*

Micro computed tomographic (µCT) determination of excised femora were carried out using the Sky Scan 1076 KCT scanner (Aartselaar, Belgium).Trabecular bone volume (BV/TV; %), trabecular separation (TbSp), trabecular number (TbN), and trabecular pattern factor were calculated by the mean intercept length method. N = 10 mice/group; data are presented as mean ± SEM;

*P<0.05,

**P<0.01,

***P<0.001 compared with Ovx+vehicle group.

**Table 2:** Thymus and spleen weight in Balb/c mice.

Parameter	Sham	Ovx	Ovx+E20.01 mg/kg/day	OVX+Daid 10 mg/kg/day
ThymusWt (mg)	48.36±3.11***	67.27±3.45	47.54±3.52***	44.87±3.14***
SpleenWt (mg)	83.10±3.13***	100.2±3.15	82.10±2.69***	82.37±3.88***

Daid treatment decreases Ovx-induced increase in thymus and spleen weight. N = 10 mice/group). Data are presented as mean ± SEM;

***P<0.001 compared with Ovx+vehicle group.

### Role of Daid on Ovx-induced ROS generation and enhanced circulating TNF-α level

**Figure 1:**
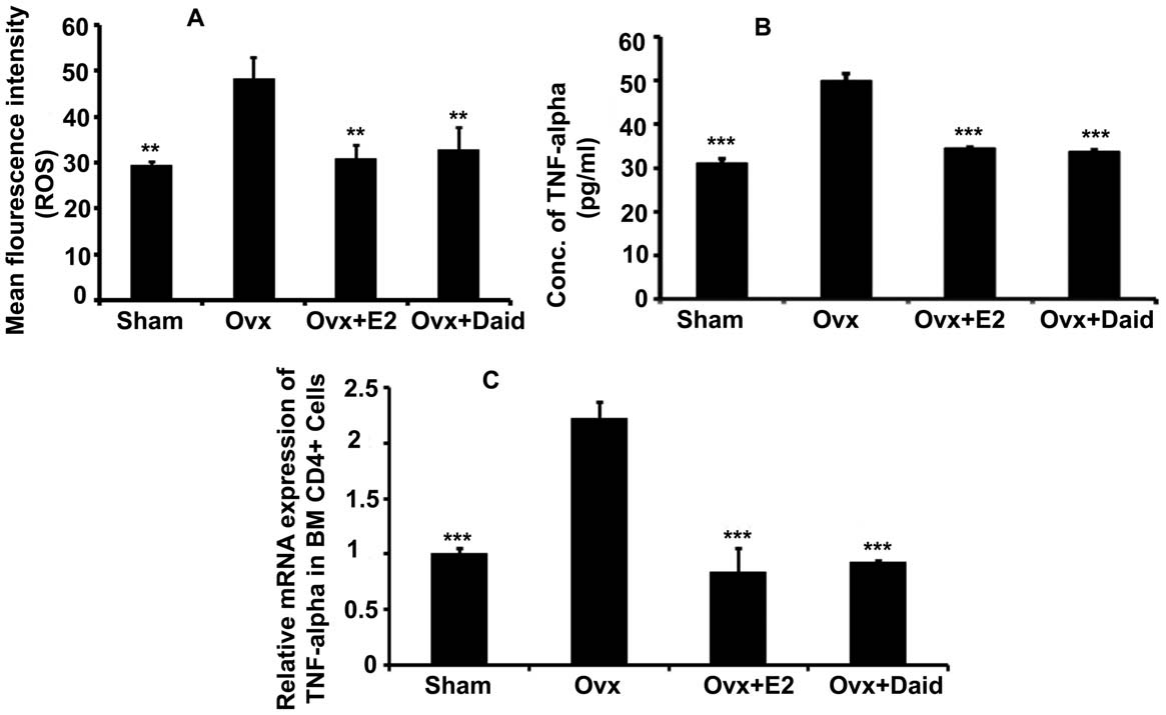
Daid treatment inhibits Ovx-induced oxidative stress in CD4^+^ T cells and TNF-α production by these cells. (A) Cellular ROS measurement was performed by incubating CD4^+^ T cells with DCF-DA followed by FACS analysis. (B) Circulating TNF-α levels were measured in various groups by ELISA. (C) TNF-α mRNA levels in the BM CD4^+^ T cells were measured in various groups by qPCR. N = 10 mice/group; data are presented as mean ± SEM; ^**^ *P*<0.01 and ^***^ *P*<0.001 compared with Ovx+vehicle group.

Ovariectomy causes an accumulation of reactive oxygen species in the BM, which leads to increased production of TNF-α by activated T cells through upregulation of the costimulatory molecule CD80 on dendritic cells [28]. Our data show that Ovx led to significant increase in ROS production compared to sham group (Fig. 1A). However, treatment with Daid led to significant reduction in ROS production (Fig. 1A). E2 deficiency is known to increase circulating TNF-α levels [1]. Our data show that Ovx mice had significantly higher circulating levels of TNF-α compared with sham group. Ovx mice treated with Daid had reduced TNF-α level that were comparable to that of sham group (Fig. 1B). Further, mice Ovx for 6 weeks had ∼2.0-fold higher TNF-α mRNA levels in BM CD4^+^ T cells compared with sham (Fig. 1C). Daid treatment of Ovx mice significantly reduced TNF-α mRNA levels compared to Ovx control group (Fig. 1C). E2 was taken as a positive control.

### Role of Daid on Ovx induced expansion of BM and Spleen CD4^+^ cells

**Figure 2:**
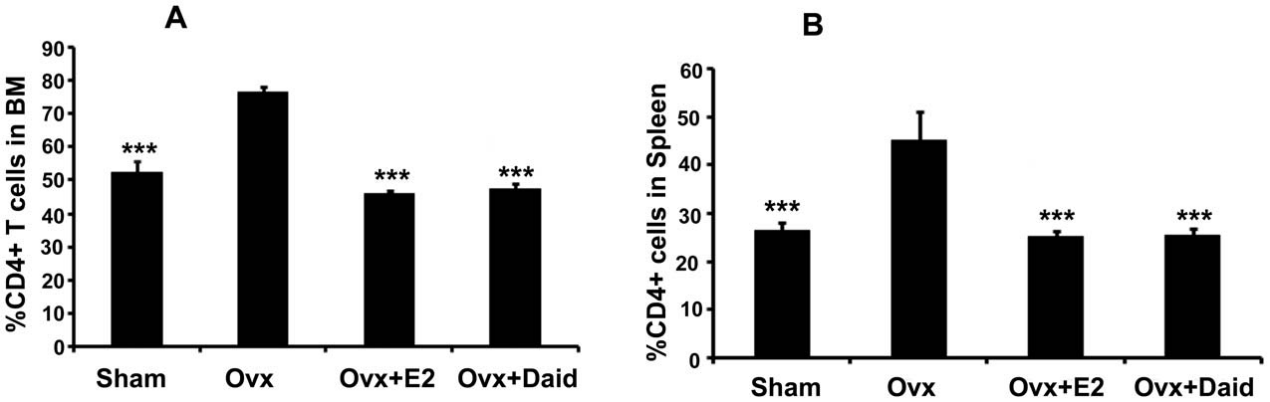
Daid treatment significantly decreased Ovx-induced increases in CD4^+^ T cell subsets in the BM and spleen. (A) CD4^+^ T cells in BM and (B) CD4^+^ T cells in spleen were quantified by flow cytometry as described in Materials and Methods. N = 10 mice/group; data are presented as mean ± SEM; ^***^ *P*<0.001 compared with Ovx+vehicle group.

Ovx is known to increase proliferation of CD4^+^ T cells [1]. Mice Ovx for 6 weeks have higher frequency of CD4^+^ T cells in BM compared with sham group (Fig. 2A). Treatment of Ovx mice with either Daid resulted in ∼50% reduction in the frequency of CD4^+^ T cells in BM compared with Ovx group (Fig. 2A). Effect of Ovx on the secondary lymphoid organ (spleen) was also studied, and we found that Daid decrease the Ovx induced expansion of CD4^+^ cells in spleen (Fig. 2B). E2 was taken as a positive control.

### Effect of Daid on Ovx-induced loss of CD28 expression in BM and Spleen T cells

**Figure 3:**
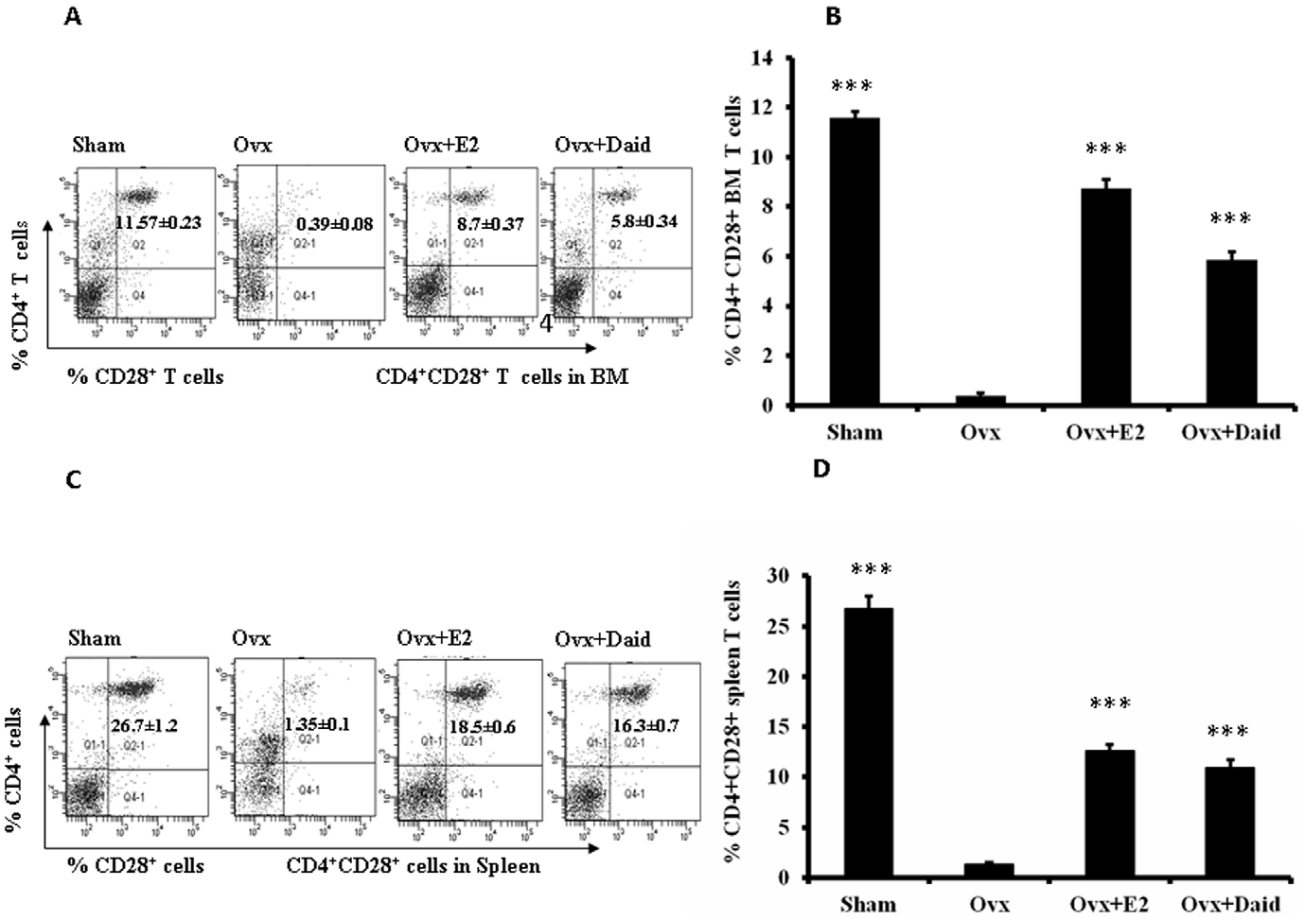
Daid treatment prevented Ovx-induced loss of CD28 on the BM as well as in spleen CD4+ T cells. (A) Representative image (B) CD4^+^CD28^+^ T cells in BM, (C) Representative image (D) CD4^+^CD28^+^ T cells in spleen of Sham, Ovx, Daid and E2 were measured by flow cytometry as described in Materials and Methods. N = 10 mice/group; data are presented as mean ± SEM; ^***^ *P*<0.001 compared with Ovx+vehicle group.

CD28 is the surface glycoprotein expressed on CD4^+^ and CD8^+^ T cells and loss of CD28 expression is an indicator of T cell senescence [5], [7]. Percentage of CD4^+^CD28^+^ T cells in BM of Ovx mice was significantly less than sham group (Fig. 3A and B). Ovx mice treated with Daid had significantly higher frequency of CD4^+^CD28^+^ T cells in BM compared with Ovx + vehicle group (Fig. 3A and B). Pattern of CD28 expression was found to be similar in Splenic CD4^+^ T cells. Daid significantly increased CD28 expression on CD4^+^ T cells compared to Ovx + vehicle group (Fig. 3C). E2 was taken as a positive control.

### Effect of Daid in co-culture of bone marrow cells and TNF-α secreting CD4^+^CD28null T cells

**Figure 4:**
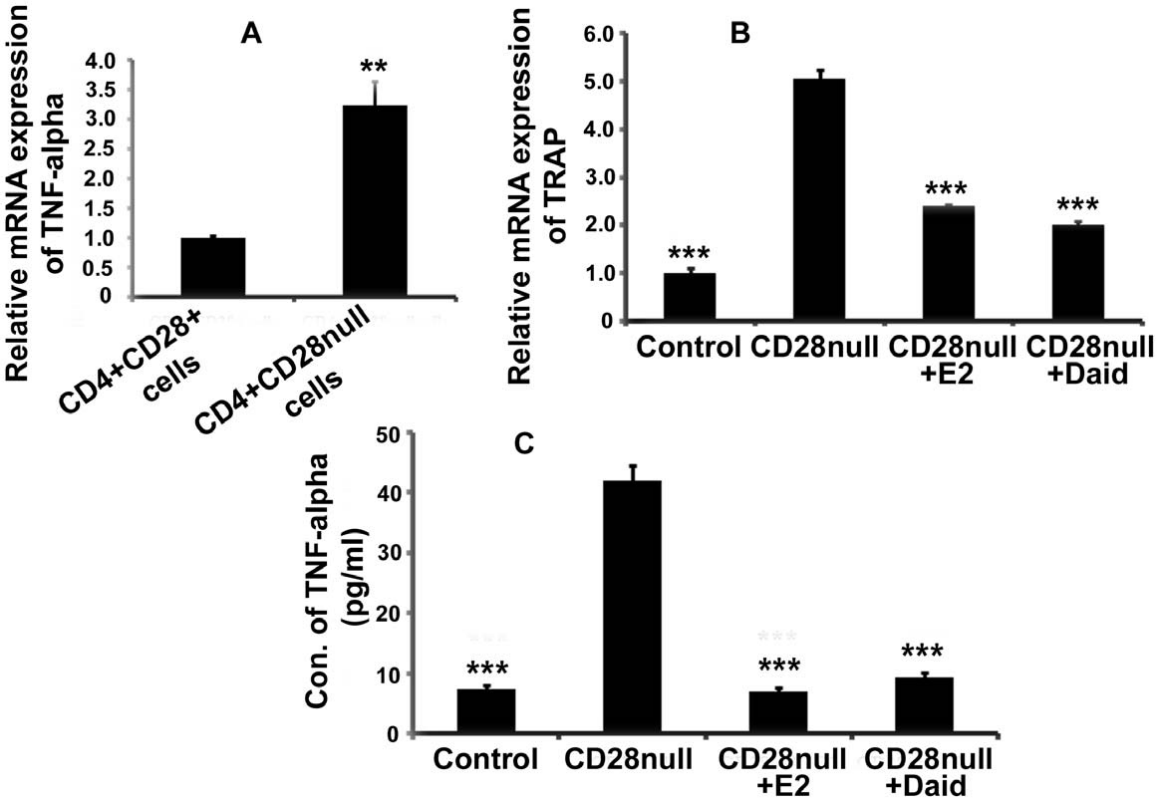
Daidzein inhibits increased osteoclastogenesis in co-culture of bone marrow cells and TNF-α secreting CD4^+^CD28null T cells. (A) TRAP mRNA expression by qPCR in co-culture of BM cells and CD4^+^CD28null T cells. N = 3 mice/group; data are presented as mean ± SEM; ^***^ *P*<0.001 compared with untreated co-culture of CD4^+^CD28null T cells with BM cells. (B) TNF-α levels were measured in the co-culture by ELISA. N = 3 mice/group; data are presented as mean ± SEM; ^***^ *P*<0.001 compared with untreated co-culture of CD4^+^CD28null T cells with BM cells.

It is established that there is an increased proliferation of TNF-α producing T cells in estrogen deficient ovx mice [1]. In an earlier study [11], we have shown that this increase in TNF-α is mainly contributed by CD4^+^CD28null T cells where TNF-α mRNA levels were several folds higher in CD4^+^CD28null T cells compared to CD4^+^CD28^+^ T cells isolated from BM. Increased TNF-α production may lead to enhanced osteoclastogenesis directly or by augmenting RANKL production [1]. Thus it was interesting to study if co-culture of CD4^+^CD28null T cells with osteoclast precursor cells may lead to enhanced osteoclastogenesis. We observed that transcript levels of TRAP were significantly higher in BM cells co-cultured with CD4^+^CD28null T cells. Treatment with Daid and E2 led to a significant decrease in the transcript levels of TRAP in CD4^+^CD28null cells co-cultured with BM cells (Fig. 4A). Conditioned media was collected for determining TNF-α level. It was observed that while TNF-α levels were higher in conditioned media of BM cells co-cultured with CD4^+^CD28null T cells, treatment with Daid and E2 decreased these levels and brought it back nearly to the basal level (Fig. 4B).

### Effect of Daid on transcript levels of nucleolin and hnRNP-D0A genes vital for CD28 gene expression

**Figure 5:**
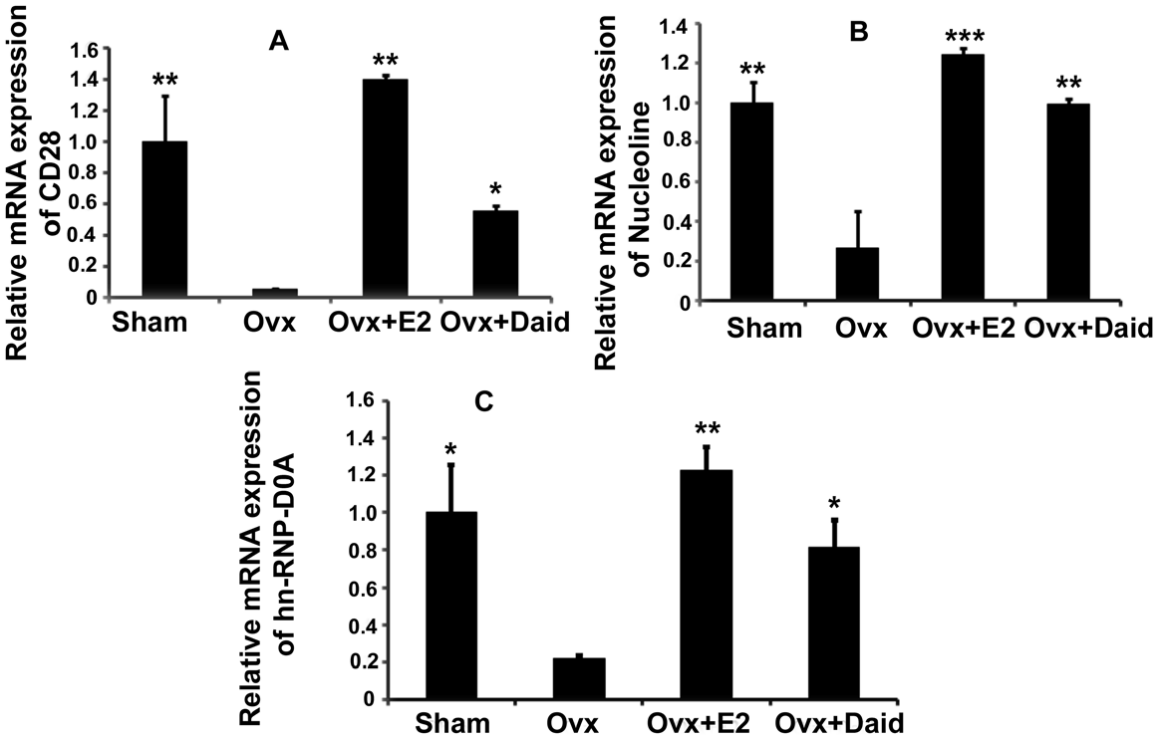
Daid treatment increased CD28, nucleolin and hnRNP-D0A mRNA levels assessed by qPCR in the BM T cells of Ovx mice. mRNA levels of CD28, nucleolin and hnRNP-D0A in CD4^+^ T cells (A–C). All data are the mean ± SD of three independent experiments; n = 3 mice/group; ^*^ *P*<0.05, ^**^ *P*<0.01, ^***^ *P*<0.001 compared with Ovx+vehicle group.

Nucleolin and hnRNP-D0A are proteins responsible for CD28 expression on T cells [7], [8]. Our data show that CD4^+^ T cells in BM of Ovx mice had significantly lower mRNA levels of CD28, nucleolin and hnRNP-D0A compared with sham group (Fig. 5A, B and C). Daid treatment of Ovx mice also led to a significant increase in mRNA levels of all the three genes i.e. CD28, nucleolin and hnRNP-D0A in CD4^+^ T cells, however less than that of Ovx+E2 treated group (Fig. 5A, B and C).

### Effect of Daid on TNF-α mediated down-regulation of CD28 on CD4^+^ BM cells *in vitro*

**Figure 6:**
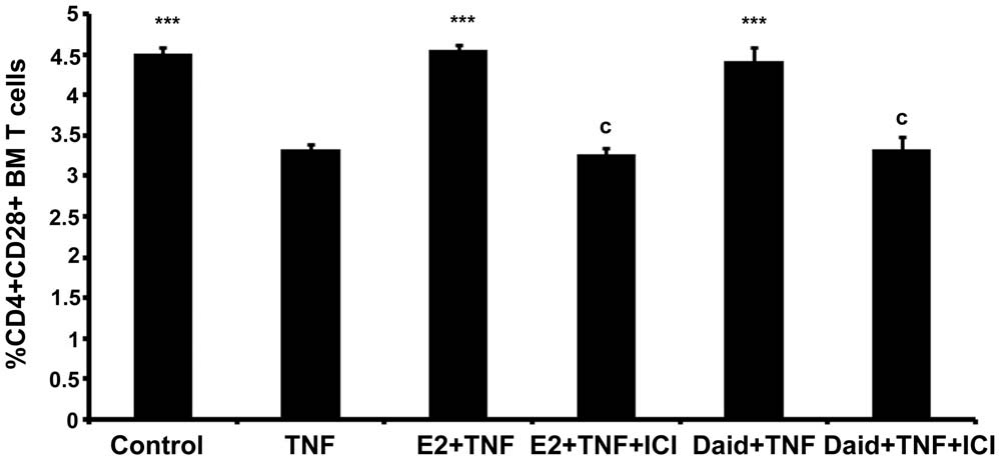
Daid abrogates TNF-α mediated CD28 downregulation in the BM CD4^+^ T cells. (A) BM CD4^+^ T cells (5×10^5^ cells/well) were seeded in 24-well plates. For ICI 182,780 (ICI) treatment group, the cells were pre-treated with ICI for 30 min. Various treatments as shown were given to CD4^+^ T cells for 24 h [E2-10^−9^ M, Daid −10^−8^ M, TNF-α−10.0 ng/ml]. At the end of incubation, cells were stained with antibodies against CD3, CD4 and CD28 and subjected to flow cytometry as described in Materials and Methods. Data are presented as mean ± SEM from 3 independent experiments; ^***^ *P*<0.001 compared with TNF-α treated cells and ^c^ *P*<0.001 compared between Daid+TNF-α and Daid+TNF-α+ICI treated cells; E2+TNF-α and E2+TNF-α+ICI treated cells.

We next studied the effect of Daid on TNF-α induced supfopression of CD28 levels in BM CD4^+^ cells and whether Daid mediated this effect via ERs. Purified CD4^+^ T cells were exposed to various treatments as shown in (Fig. 6) and CD28^+^ T cells were quantified by flow cytometry. Exogenous TNF-α significantly reduced the frequency of CD28^+^ T cells, while Daid attenuates TNF-α induced reduction of CD28^+^ T cells (Fig. 6). Presence of an anti-estrogen, ICI-182,780, blunted the ability of Daid to attenuate TNF-α induced reduction of CD28^+^ T cells.

### Role of Daid in Ovx induced elevation of B-lymphopoesis

**Figure 7:**
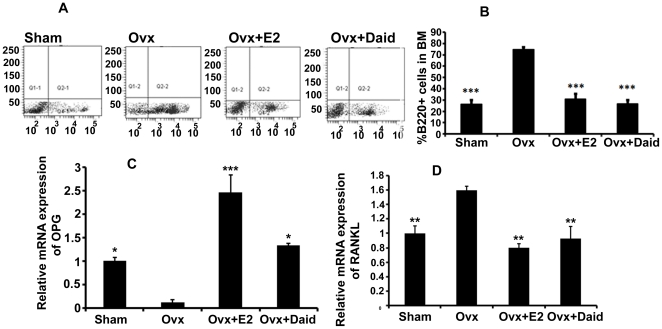
Daid treatment significantly decreased Ovx-induced increases in B220^+^ cells in BM. (A and B) B220^+^ cells in BM were quantified by flow cytometry as described in Materials and Methods. (C) OPG mRNA levels in the BM B220^+^ cells were measured in various groups by qPCR. (D) RANKL mRNA levels in the BM B220^+^ cells were measured in various groups by qPCR. N = 10 mice/group; data are presented as mean ± SEM; ^*^ *P*<0.05, ^**^ *P*<0.01, ^***^ *P*<0.001 compared with Ovx+vehicle group.

Ovx is known to increase the proliferation of B220^+^ cells in bone marrow while estrogen treatment restores the increased level of B-lymphopoiesis in Ovx mice to the sham level [25]. It was seen that mice Ovx for 6 weeks have higher frequency of B220^+^ cells in BM compared with sham group (Fig. 7A and B). Treatment of Ovx mice with Daid resulted in more than 50% reduction in the frequency of B220^+^ cells in BM compared with Ovx group (Fig. 7A and B). E2 was taken as a positive control.

Additionally, estrogen deficiency upregulates the production of RANKL from B cells [29] which is an important factor for osteoclastogenesis. B cells are also a source of osteoprotegrin, decoy receptor for RANKL [30]. It was seen that in B220^+^ cells isolated from Ovx mice, the transcript levels of OPG were very low compared to those isolated from sham group (Fig. 7C). Treatment of Ovx mice with Daid led to an increase in OPG levels however, E2 effect was more robust (Fig. 7C). Mice Ovx for 6 weeks show increased transcript levels of RANKL compared to sham group in B220^+^ cells (Fig. 7D). Treatment of Ovx mice with Daid led to a decrease in RANKL transcript levels (Fig. 7D).

## Discussion

Daid (10.0 mg.kg^−1^.day^−1^ dose) or E2 (0.01 mg.kg^−1^.day^−1^ dose) given for 6 weeks to Ovx mice protected against Ovx-induced loss of trabecular microarchitecture (Table 1). At this osteoprotective dose of Daid, we demonstrate that Daid reverses several Ovx-induced changes including 1) increased thymic and spleen weight, 2) increased TNF-α expression in BM CD4^+^ T cells, 3) increased circulating TNF-α levels, 4) increased population of BM and Spleen CD4^+^ T cells, 5) reduced CD28 expression on BM and Spleen CD4^+^ T cells, 6) increased B-lymphopoesis in BM cells.

Ovx is known to increase the thymic and spleen weight which contributes to increased thymic T cell output and B cell maturation leading to more bone loss [26], [31]. Genistein has been shown to cause thymus involution in mice [32], [33]. Previous reports have shown that while E2 treatment led to 50% decrease in thymus weight, genistein at a dose of 80 mg/kg and 200 mg/kg body weight dose led to a drastic decrease (73% and 78% decrease respectively) in thymus weight suggesting the possibility of thymic abnormalities [32]. In our case Ovx mice were treated with 10 mg/kg body weight dose of Daid which led to 40% reduction in thymus weight, almost similar to E2 treated Ovx mice and thus appears to be safe (Table 2). Daid also reduced Ovx induced gain in spleen weight (Table 2).

Reports have suggested that stimulation of TNF-α production by osteoclasts or BM cells is the mechanism by which ROS cause bone loss [34]. Studies by Grassi et al. [28] have shown that key effects of Ovx, the up-regulation of Ag-dependent activation of T cells and the resulting T cell production of TNF-α, are mediated by ROS and abolished by treatment with antioxidants. Our data show that while Ovx led to significant increase in ROS production by CD4^+^ T cells, treatment with Daid inhibited Ovx induced ROS generation (Fig. 1A). We further observed that the circulating levels of TNF-α were ∼60% higher in Ovx mice compared with sham, which is in agreement with previous reports [35], and Daid treatment of Ovx mice totally obliterated Ovx-induced increase in circulating TNF-α levels (Fig. 1B). Also, TNF-α mRNA levels were elevated by >2.0-fold in BM CD4^+^ T cells of Ovx mice compared with sham, and Daid treatment completely abolished Ovx-induced upregulation of TNF-α mRNA levels (Fig. 1C). Together, our data suggest that Daid alleviates systemic and local (via BM T cells) rises in TNF-α caused by E2 deficiency and thus prevent bone wasting.

Similar to that in the thymus, Ovx causes an expansion of the T cell pool in BM (Fig. 2A) and spleen (Fig. 2B) by increasing T cell proliferation and lifespan [1], [36]. We observed that Daid reduced the proportion of Ovx-induced increases in BM (Fig. 2A) and spleen CD4^+^ T cells (Fig. 2B). Additionally, Daid completely mitigated Ovx-induced loss of CD28 on CD4^+^ T cells of the BM and spleen. According to inflammation theory of aging, T cells deficient in CD28 expression become prematurely senescent and acquire the ability to produce pro-inflammatory cytokines. In mammals, E2 deficiency is an aging process, signalling the end of reproductive life [37]. We observed that Ovx resulted in loss of CD28 expression in BM and spleen T cells (Fig. 3). Supplementing Ovx mice with Daid restored the percentage of CD28^+^ T cells in both BM and spleen cells (Fig. 3). These data suggest that Ovx triggers T cell senescence in the BM as well as in spleen by reducing CD28 expression and Daid counteracts this loss.

Our earlier study [11] has shown that increased mRNA levels of TNF-α is mainly contributed by CD4^+^CD28null T cells. As TNF-α directly or by augmenting RANKL secretion may lead to enhanced osteoclastogenesis it was studied if CD4^+^CD28null T cells co-cultured with osteoclast precursor cells lead to increased osteoclast differentiation. We found that TRAP mRNA expression was significantly more in CD4^+^CD28null T cells co-cultured with BM cells compared to control and treatment with Daid and E2 decreased TRAP transcript levels in the co-culture (Fig. 4B). Moreover, TNF-α levels when determined by ELISA in conditioned media of CD4^+^CD28null T cells co-cultured with BM cells were higher and were decreased after Daid and E2 treatment (Fig. 4C).

Next, we determined the mode by which Daid inhibits CD28 loss as a result of ovariectomy. Basal transcription of CD28 gene is regulated by nucleolin-hnRNP-D0A-site α complex formation in the CD28 promoter [7], [8]. Daid treatment of Ovx mice caused significantly greater increase in the levels of CD28 mRNA with E2 showing a more robust effect. Also, while nucleolin and hnRNP-D0 mRNA levels were significantly reduced in CD4^+^ T cells in Ovx mice compared with sham group, treatment of Ovx mice with Daid significantly enhanced the transcript levels of nucleolin and hnRNP-D0 mRNA levels in BM CD4^+^ T cells (Fig. 5). These data suggest that Daid prevent loss of CD28 expression by inhibiting the reduction in the mRNA levels of nucleolin and hnRNP-D0A in CD4^+^ T cells as a result of ovx.

There is evidence that TNF-α down regulates CD28 expression on T cells leading to the emergence of senescent cell population [38]. We show that treatment of exogenous TNF-α to BM CD4^+^ T cells resulted in reduced percentage of CD28^+^ T cells compared with vehicle treated CD4^+^ T cells, and the presence of Daid with TNF-α significantly reversed TNF-α-induced loss of CD28. Further, presence of ICI-182,780 blocked the ability of Daid to reverse TNF-α induced loss of CD28 on T cells (Fig. 6). Thus, we hypothesize that Daid might be acting via the ERs to abrogate TNF-α-induced loss of CD28 expression in T cells. This would result in decreased production of senescent T cell population that in turn may be responsible for increased TNF-α production under E2 deficiency.

It has been reported that estrogen deficiency caused by Ovx selectively stimulates B-lymphopoiesis, resulting in an accumulation of pre-B cells in mouse bone marrow [39]. It was assumed that the increased B-lymphopoiesis caused by estrogen deficiency was involved in stimulating bone resorption [12]. Studies on the isoflavone genistein have shown that it reduces B-lymphopoesis [25]. Thus, it was studied if Daid also reduces B-lymphopoesis as is the case with genistein. Our data show that while Ovx led to significant increase in percent of B220^+^ cells in BM compared to sham, treatment with Daid inhibited this proliferation of B220^+^ and brought it back to nearly the sham level (Fig. 7A and B). Mature B cells have the potential to both positively and negatively impact osteoclastogenesis by secreting pro-osteoclastogenic cytokines like RANKL, as well as anti-osteoclastogenic cytokines such as OPG [13]. RANKL/RANK/OPG represents the triad in the cross-talk between osteoblasts and osteoclasts that regulates osteoclastogenesis [40]. Thus, the effect of Daid on OPG and RANKL mRNA transcripts in B cells was observed. B cells isolated from Ovx mice and treated with Daid exhibited increased OPG mRNA level (Fig. 7C) and decreased RANKL mRNA levels (Fig. 7D) compared with Ovx control mice, with E2 showing much robust effect.

To conclude, our results reveal that Daid reverses detrimental immunological changes resulting from E2 deficiency provides protection against bone loss. Based on our findings, we propose that Daid prevents bone loss by (i) inhibiting the proliferation of TNF-α producing CD4^+^CD28null T cells, (ii) preventing premature T cell senescence via increasing mRNA levels of nucleolin, hnRNP-D0A, and CD28 in BM T and by antagonizing TNF-α-induced loss of CD28 expression via ER and (iii) by inhibiting Ovx induced B-lymphopoesis.

## Materials and Methods

### Reagents and chemicals

Mouse lymphocytes from bone marrow and spleen were cultured in complete RPMI-1640 medium (Wisent Inc., St-Bruno, QC, Canada) supplemented with 10% fetal bovine serum (FBS), Penicillin (500 U/ml), Streptomycin (500 mg/ml). Trizol was purchased from GIBCO-BRL, Invitrogen corp., Carlsbad, CA; TNF-α and DCF-DA from Sigma-Aldrich (St. Louis, MO, USA). RPE-cy-7, PE-cy-5.5-conjugated anti-mouse CD4, FITC conjugated anti-mouse CD28 and APC conjugated anti-mouse B220^+^ antibodies were purchased from BD Biosciences (Mississauga, ON, CA). CD4 (L3T4), CD28, and B220^+^ microbeads were purchased from Miltenyi Biotech, Germany and TNF-α ELISA kit from Immunodiagnostic systems Ltd. UK.

### *In vivo* study

The study was conducted in accordance with current legislation on animal experiments (Institutional Animal Ethical Committee at Central Drug Research Institute) and was approved by the Institutional animal ethics committee. Approval ID for this study was 39/10/ENDO/IAEC dated 22.1.2010. Adult Balb/c mice (9–10 week-old) were taken for the study [41], [42], [43], [44]. All mice were housed at 25°C, in 12-hour light∶12-hour dark cycles. Normal chow diet and water were provided ad libitum. Ten mice per group were taken for the study, and all rats in each group were assayed and included in the statistical analyses (n = 10). The groups were as follows: sham-operated (ovary intact) mice, which served as the control group and were given vehicle (gum acacia in distilled water); Ovx+vehicle; Ovx+0.01 mg.kg^−1^.day^−1^ E2 [45], [46]; and Ovx+10 mg.kg^−1^.day^−1^ daidzein (Daid) [47]. All treatments were given by oral gavage and continued for 6 weeks. At the completion of study, animals were autopsied. After autopsy, bones were dissected and the bone marrow (BM) was flushed out. Total lymphocytes from the BM were isolated by using Hisep LSM 1084 (Himedia) by means of density (1.084±0.0010 g/ml) gradient centrifugation technique [48], [49]. Pure CD4^+^, CD4^+^CD28^+^ and CD4^+^CD28null T and B220^+^ cells were retrieved from the BM by positive and negative selection using microbeads based isolation by MACS separator according to the manufacturer's protocol (EasySep Biotin Selection Kit, Stem Cell Technologies Inc., Vancouver, BC, CA). These purified cells were then collected in Trizol for Real time PCR (qPCR). Thymuses and spleens were collected and weight recorded. Serum was collected for ELISA. Serum TNF-α was measured in all the groups by using ELISA kit according to manufacturer's instructions.

### Flow cytometry

Cells from the BM/Spleen were labelled with anti CD3, CD4 , CD28 and B220 antibodies (APC conjugated anti-mouse CD4, PE-cy-5.5-conjugated anti-mouse CD4, FITC conjugated anti-mouse CD28 and APC conjugated anti B220 antibodies) to assess the percentage of CD4^+^, CD4^+^CD28^+^ in CD3^+^ cells and B220^+^ (CD45RO) cells. Specificity of immunostaining was ascertained by the background fluorescence of cells incubated with Ig isotype controls. Fluorescence data from at least 10,000 cells were collected from each sample. Immunostaining was done as per manufacturer's instructions. In brief, single cell suspension of the BM was prepared in PBS. Then cells were centrifuged at 500 rpm for 5 minutes. Supernatant was discarded and cells were suspended in 1 ml of PBS. Cells were counted using haemocytometer and were resuspended in PBS as 10^6^ cells/100 µl PBS and antibody were added as 10 µl/10^6^ cells and further incubated for 45 minutes at room temperature. After incubation cells were washed twice with PBS and transferred to FACS tubes for analysis. FACS Caliber and FACS Arya (BD Biosciences Mississauga, ON, CA) were used to quantify the percentage of CD4^+^, CD4^+^CD28^+^ T cells in CD3^+^ cells and B220^+^ B cells in all the groups [50].

### Bone Marrow- CD4^+^CD28null T cell Co-culture

For bone marrow-T cell co-culture, bone marrow cells (BMCs) were isolated from 4- to 6 week old mice by flushing bone marrow from the excised femora with osteoclast cell culture medium containing 10% FCS. Cells were cultured overnight in osteoclast culture medium containing macrophage-colony stimulating factor (MCSF) (7 ng/ml). After overnight incubation, non-adherent bone marrow cells were added to purified CD4^+^CD28null T cells in osteoclast culture medium containing M-CSF (30 ng/ml) and receptor activator of nuclear factor kappa-B ligand (RANKL) (50 ng/ml). After six days in culture, cells were harvested for RNA and cDNA synthesis. Transcript level of tartarate resistant acid phosphatase (TRAP), an osteoclastogenic marker, was determined by q-PCR. Conditioned media was collected for TNF-α ELISA.

### Total RNA isolation and quantitative Real-Time-PCR

Total RNA was extracted from isolated CD4^+^, CD4^+^CD28^+^ and CD4^+^CD28null T and B220^+^ cells of all the *in vivo* groups and *in vitro* cultured cells using Trizol (Invitrogen). cDNA was synthesized from 1 µg total RNA with the Revert AidTM H Minus first strand cDNA synthesis kit (Fermentas, USA). SYBR green chemistry was used for quantitative determination of the mRNAs for CD28, Nucleolin, hn-RNP-D0A, OPG, RANKL, TRAP, TNF-α and a housekeeping gene, GAPDH, following an optimized protocol. The design of sense and antisense oligonucleotide primers was based on published cDNA sequences using the Universal probe library (Roche Diagnostics, USA). Primer sequences are given in Table 3. For real-time PCR, the cDNA was amplified with Light Cycler 480 (Roche Diagnostics Pvt. Ltd.).

**Table 3:** Sequences of real-time PCR primers.

Gene Name	Primer Sequence	Accession Number
**CD 28**	P1-5′-CTGGCCCTCATCAGAACAAT-3′P2-5′-GGCGACTGCTTTACCAAAATC-3′	NM_007642.4
**Nucleolin**	P1-5′-CATGGTGAAGCTCGCAAAG-3′P2-5′-TCACTATCCTCTTCCACCTCCTT-3′	NM_010880.3
**hn-RNO-D0A**	P1-5′-CAAGATCGACGCCAGTAAGA-3′P2-5′-GTGTCGTGGGGAGGAGTTT-3′	NM_001077266.1
**OPG**	P1-5′-GTTTCCCGAAGGACCACAAT-3′P2-5′-CCATTCAATGATGTCCAGGAG-3′	U94331.1
**RANKL**	P1-5′- TGAAGACACACTACCTGACTCCTG-3′P1-5′- CCACAATGTGTTGCAGTTCC-3′	AF019048.1
**TNF-α**	P1-5′-TCTTCTCATTCCTGCTTGTGG-3′P2-5′-GGTCTGGGCCATAGAACTGA-3′	NM_013693.2
**TRAP**	P1-5′-GGTCAGCAGCTCCCTAGAAG-3′P2-5′-GGAGTGGGAGCCATATGATTT-3′	NM_001102405.1
**GAPDH**	P1-5′- AGCTTGTCATCAACGGGAAG -3′P2-5′- TTTGATGTTAGTGGGGTCTCG -3′	NM_008084.2

The double-stranded DNA-specific dye SYBR Green I was incorporated into the PCR buffer provided in the Light Cycler 480 SYBER green I master (Roche Diagnostics Pvt. Ltd.) to allow for quantitative detection of the PCR product in a 20 µl reaction volume. The temperature profile of the reaction was 95°C for 5 min, 40 cycles of denaturation at 94°C for 2 min, and annealing and extension at 62°C for 30 sec, extension at 72°C for 30 sec. GAPDH was used to normalize differences in RNA isolation, RNA degradation, and the efficiencies of the reverse transcription.

### *In vitro* culture of CD4^+^ cells for TNF-α induced CD28 loss

For this experiment, lymphocytes were isolated by using Hisep LSM 1084 (Himedia) according to manufacturer's instructions. Pure CD4^+^ cells were isolated via MACS by using CD4^+^ magnetic beads according to standard protocol. After isolation cells were seeded overnight in 48 well plates at 5×10^5^cells/well in 10% RPMI 1640 media. Cells were incubated Daid (10^−8^ M) and 10^−8^ M E2 followed by incubation with TNF-α (10 ng/ml) for 24 h at 37°C. Cells were then incubated with FITC conjugated anti- CD28, PE-Cy5.5 conjugated anti CD4 and APC conjugated anti-CD3 antibody for 30 minutes in dark. After incubation cells were pelleted down and supernatant was discarded. Cells were washed in PBS. After washing cells were transferred in FACS tubes and total CD4^+^ cells were analysed in CD3^+^ cells by FACS for CD28 expression [51].

For inhibitor studies cells were pre-treated with ICI-182780 (Tocris Bioscience, USA) at 10^−9^ M concentration thirty minutes prior to Daid/E2 and TNF-α treatment.

### *Ex vivo* culture of CD4^+^ T cells for ROS generation

For *ex vivo* studies animals were autopsied and total lymphocytes were isolated from bone marrow of long bone (femur) of animals by density gradient centrifugation. Pure CD4^+^ cells were isolated by MACS using strandard protocols according to manufacturer's instructions. Purified cells were seeded in 48 well plates at a density of 3×10^5^cells/well for 24 h in 1% RPMI-1640 medium. For ROS measurement, cells were incubated with 2′ 7′ -Dichlorofluorescin diacetate DCF-DA (10 µg/ml concentration) for 30 minutes. After incubation cells were pelleted and supernatant was discarded. Cells were washed in PBS. After washing cells were transferred in FACS tubes and analysed by FACS for ROS generation [51].

### Statistical Analysis

Data are expressed as mean ± S.E.M. The data obtained in experiments with multiple treatments were subjected to one-way ANOVA followed by Newman–Keuls test of significance using Prism version 3.0 software. Student's t-test was used to study statistical significance in experiments with only two treatments. Qualitative observations have been represented following assessments made by three individuals blinded to the experimental designs.
